# Trends in cerebrospinal fluid leak rates following the extended endoscopic endonasal approach for anterior skull base meningioma: a meta-analysis over the last 20 years

**DOI:** 10.1007/s00701-020-04641-x

**Published:** 2020-11-14

**Authors:** Amir H. Zamanipoor Najafabadi, Danyal Z. Khan, Ivo S Muskens, Marike L. D. Broekman, Neil L. Dorward, Wouter R. van Furth, Hani J. Marcus

**Affiliations:** 1 Department of Neurosurgery, University Neurosurgical Centre Holland, Leiden University Medical Centre, Haaglanden Medical Center and Haga Teaching Hospital, Leiden and The Hague, The Netherlands; 2 Department of Neurosurgery, National Hospital for Neurology and Neurosurgery, London, UK; 3 Wellcome/EPSRC Centre for Interventional and Surgical Sciences, University College London, London, UK

**Keywords:** Meningioma, Endoscopic surgery, Skull base, Cerebrospinal fluid leak

## Abstract

**Objective:**

The extended endoscopic approach provides unimpaired visualization and direct access to ventral skull base pathology, but is associated with cerebrospinal fluid (CSF) leak in up to 25% of patients. To evaluate the impact of improved surgical techniques and devices to better repair skull base defects, we assessed published surgical outcomes of the extended endoscopic endonasal approach in the last two decades for a well-defined homogenous group of tuberculum sellae and olfactory groove meningioma patients.

**Methods:**

Random-effects meta-analyses were performed for studies published between 2004 (first publications) and April 2020. We evaluated CSF leak as primary outcome. Secondary outcomes were gross total resection, improvement in visual outcomes in those presenting with a deficit, intraoperative arterial injury, and 30-day mortality. For the main analyses, publications were pragmatically grouped based on publication year in three categories: 2004–2010, 2011–2015, and 2016–2020.

**Results:**

We included 29 studies describing 540 patients with tuberculum sellae and 115 with olfactory groove meningioma. The percentage patients with CSF leak dropped over time from 22% (95% CI: 6–43%) in studies published between 2004 and 2010, to 16% (95% CI: 11–23%) between 2011 and 2015, and 4% (95% CI: 1–9%) between 2016 and 2020. Outcomes of gross total resection, visual improvement, intraoperative arterial injury, and 30-day mortality remained stable over time

**Conclusions:**

We report a noticeable decrease in CSF leak over time, which might be attributed to the development and improvement of new closure techniques (e.g., Hadad-Bassagasteguy flap, and gasket seal), refined multilayer repair protocols, and lumbar drain usage.

**Supplementary Information:**

The online version contains supplementary material available at 10.1007/s00701-020-04641-x.

## Introduction

In the last two decades, the limits of endoscopic endonasal skull base surgery have been investigated. Current extended approaches allow exposure of the area between the olfactory groove and the odontoid process for resection of different pathologies (e.g., meningioma and chordoma) [28, 31]. Originally used for transsphenoidal surgery of sellar pituitary tumors, resection of tuberculum sellae meningioma, and later of olfactory groove meningioma were intuitive steps in the evolution of the extended endoscopic approach [6, 13, 15].

With the addition of the extended endoscopic approach to the arsenal of the surgeon, certain tumors can be approached from below with unimpaired visualization and direct access to the pathology, with minimal exposure and manipulation of unaffected critical neurovascular structures [26]. In patients with tuberculum sellae and olfactory groove meningioma, there is evidence that in selected patients the endoscopic approach results in better visual outcomes compared with the transcranial approach with overall low complication rates [22]. However, these extended approaches result in large dural defects and an increased risk of cerebrospinal fluid (CSF) leak in up to 25% of patients [22]. In order to address this risk, various techniques to prevent CSF leaks have been described and optimized by surgeons over the years, using lumbar drains and based on the principle of multilayer closure with autologous and synthetic materials [25, 34]. Landmark developments were the description of the vascularized pedicled Hadad-Bassagasteguy flap, its modification to a “rescue flap”, and more recently the gasket seal closure technique [11, 18, 30]. As these extended approached are still relatively new and are used for uncommon pathologies, a learning curve has been described by multiple groups [17, 33].

To evaluate the impact of these modifications and a possible learning curve, we evaluated outcomes of the extended endoscopic endonasal approach in the last two decades for a well-defined homogenous group of patients with tuberculum sellae and olfactory groove meningioma in terms of CSF leak and other surgical outcomes using a meta-analyses approach.

## Methods section

### Article selection and data extraction

A previously published literature search in Pubmed and Embase considering publications after 2004 (first paper) on outcomes of tuberculum sellae and olfactory groove meningioma patients operated with the extended endoscopic and transcranial approach was updated on 19 April 2020 [22]. Details of this search strategy are provided in the original publication [22]. Articles eligible for the current analyses were studies describing original data of the extended endoscopic approach in at least 5 patients, and articles were excluded describing a combined surgical approach, a pediatric patient population (< 18 years old), or outcomes of reoperations. The following data points were extracted from each publication: publication year, study period, study size, mean or median age, tumor location, and the outcomes of interest: number of patients with gross total meningioma resection, improvement in visual outcomes in those with preoperative deficits, CSF leak, intraoperative arterial injury, and all-cause 30-day mortality.

### Risk of bias assessment

We have adapted the New-Castle Ottawa Scale for risk of bias assessment. This scale is scored out of 6 and assesses sample selection, outcome reporting, and comparability between treatment arms. As no comparative studies were assessed in our study, we omitted the latter domain.

### Main analyses

For the main analyses, we pragmatically grouped publications based on publication year in three categories (2004–2010, 2011–2015, and 2016–2020). As in earlier years, fewer publications were published; we chose the first category to span a year longer than the other categories. We evaluated the percentage patients with a CSF leak as primary outcome. Secondary outcomes were the percentage patients with a gross total meningioma resection, improvement in visual outcomes in those with preoperative deficits, intraoperative arterial injury, and 30-day mortality.

### Sensitivity analyses

We also performed multiple sensitivity analyses to assess the robustness of the results and the possible effects of information bias, classification bias, and selection bias.

First, as publications from the same year might cover different study periods, we categorized studies in three categories based on the median calendar year of the described study period: 2000–2005, 2006–2010, and 2011–2015.

Second, we performed analyses separately for patients with tuberculum sellae meningioma and olfactory groove meningioma. Although the analyses with only patients with olfactory groove meningioma should be interpreted with caution, as the number of studies and patients within some analyses is very small.

Third, we compared publications that specifically described routine use of pedicled nasoseptal flaps (e.g., Hadad-Bassagasteguy flap) with those that did not describe routine use of these flaps*.* No other comparisons were made concerning closure techniques, due to paucity of data on other well-defined techniques.

Fourth, we compared publications that specifically described the routine use of lumbar drains to prevent CSF leaks with those that did not describe routine use of lumbar drains*.*

### Used statistics

Random-effect estimates with 95% confidence intervals were calculated using the DerSimonian and Laird method [8]. A Freeman-Tucky double arcsine transformation was performed to include studies with 0% or 100% outcomes [24]. *I*^2^ statistics were used for quantification of between-study heterogeneity. If multiple patient groups (e.g., patients with tuberculum sellae and olfactory groove meningioma) were described separately within one publication, each group was entered separately in the analyses to account for the heterogeneity between the groups with the use of the random-effects model. No formal statistics were assessed to obtain *p* values for the performed comparisons, as none of the comparisons were described in the original studies. Comparison of different patient groups could be strongly affected by differences in patient and tumor characteristics, which are often confounders for the comparisons. Instead results are reported for each group, including 95% confidence intervals (95%CI), describing the accuracy of the aggregated results within the group [10]. Publication bias was assessed by generating a funnel plot for the main analyses with and without the Duval and Tweedie trim-and-fill method [9]. Analyses were performed with Stata version 16.1 (Statacorp).

## Results section

### Study characteristics

**Fig. 1:**
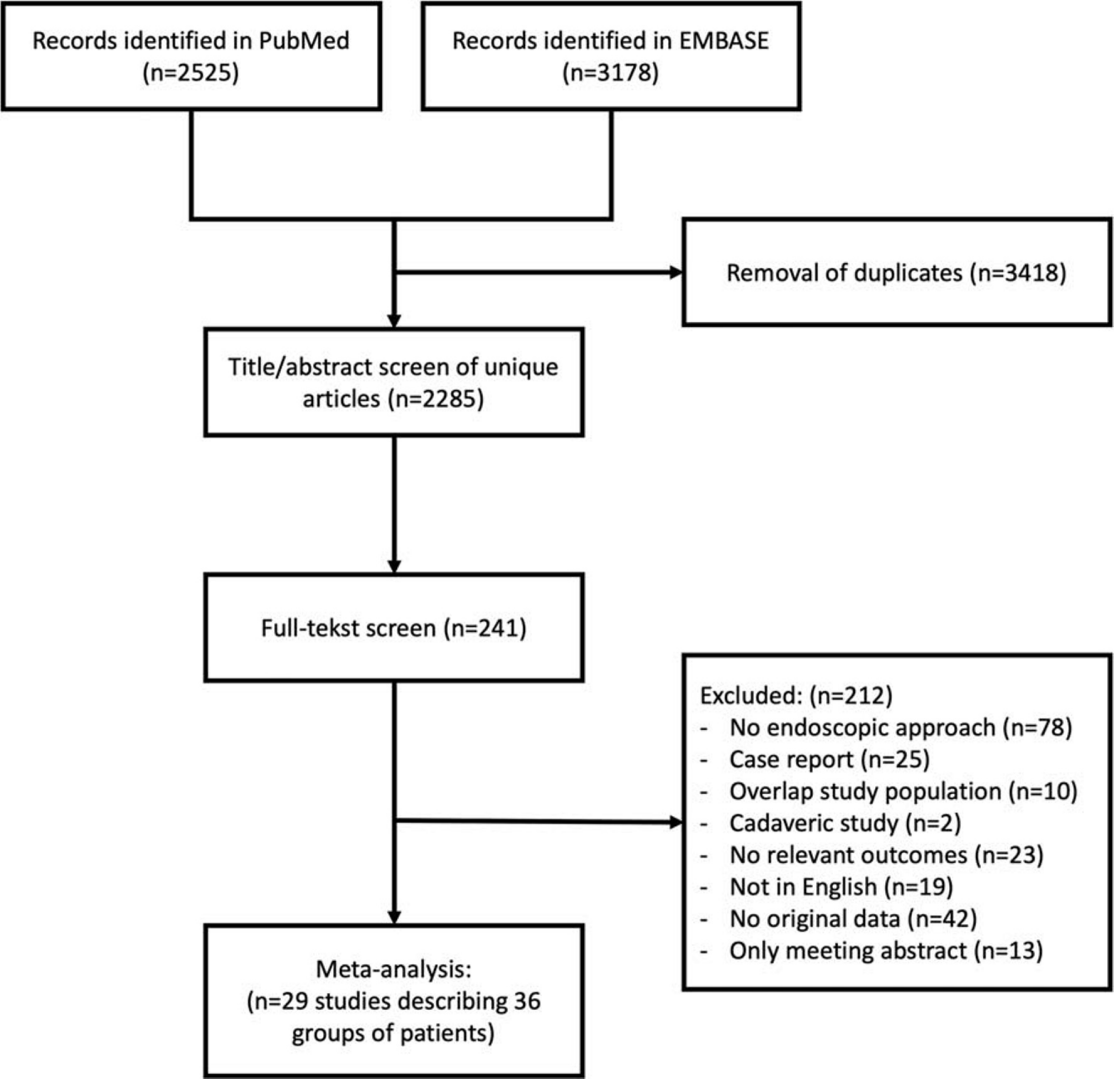
Flow chart of article screening and selection

A total of 2285 articles were screened for title and abstract, and of 241 articles, the full text was read to assess eligibility. We eventually included 29 studies describing 36 groups of patients consisting of 540 patients with tuberculum sellae meningioma patients and 115 with olfactory groove meningioma (Fig. 1 and Supplementary Table 1). The median age was 54 years (interquartile range (IQR): 52–59), and the median percentage of male patients included was 24% (IQR: 14–33%). Risk of bias scores for individual studies is depicted in Supplementary Table 1. Four studies (11%) were classified as low risk of bias on both sample selection and outcome reporting. Fifteen studies (42%) scored low risk of bias only on sample selection and five (8%) only on outcome reporting.

### Trends over time

**Fig. 2:**
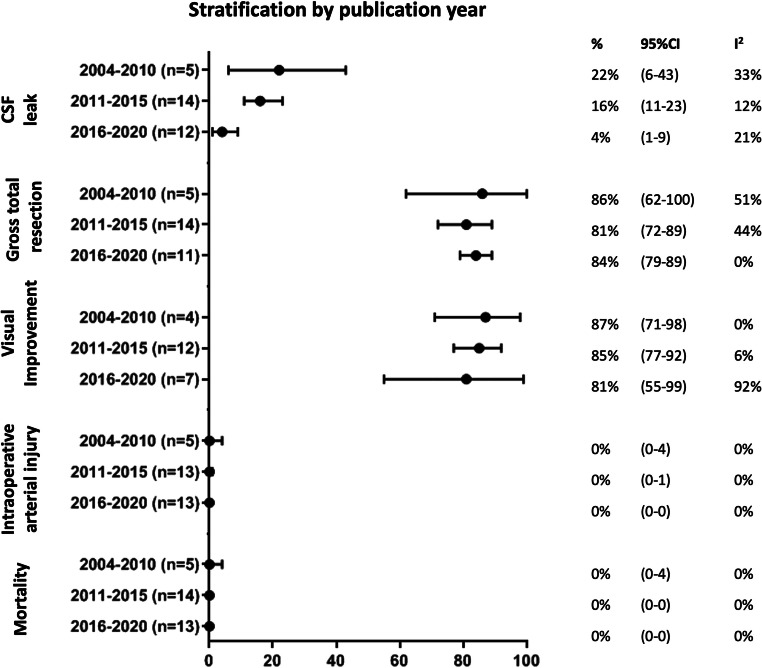
Outcomes stratified by publication year

The percentage patients with a CSF leak dropped over time from 22% (95% CI: 6–43%) in studies published between 2004 and 2010, to 16% (95% CI: 11–23%) between 2011 and 2015, and 4% (95% CI: 1–9%) between 2016 and 2020 (Fig. 2). Outcomes of gross total resection, visual improvement, intraoperative arterial injury, and 30-day mortality remained stable over time (Fig. 2). Impact of publication bias was limited for these outcomes, as there was limited asymmetry in the funnel plots without any major change in effect estimates using the trim and fill method (Supplementary Figure 1).

**Fig. 3:**
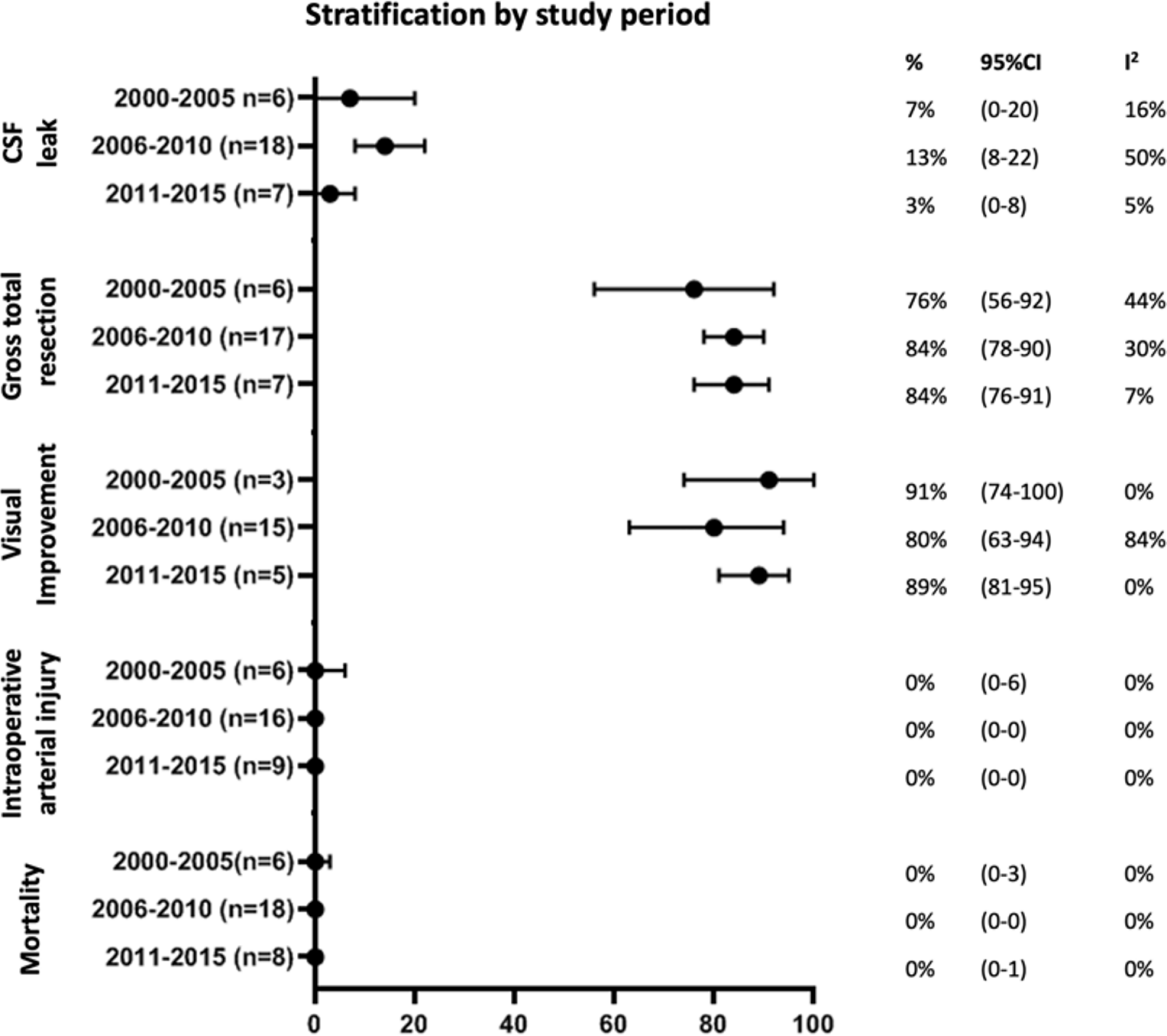
Sensitivity analyses: outcomes stratified by study period (median calendar year of reported study period)

**Fig. 4:**
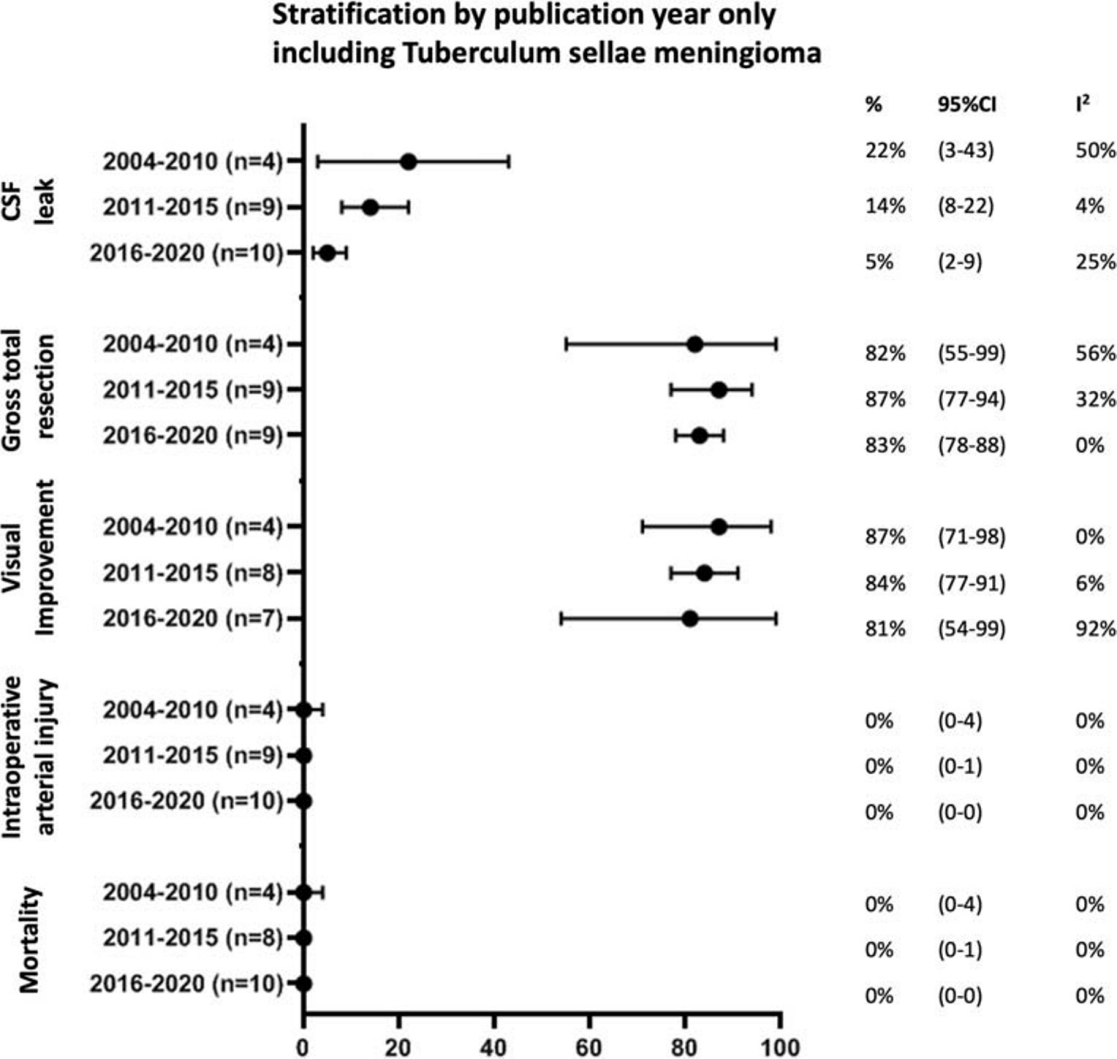
Sensitivity analyses: outcomes stratified by publication year, only including patients with tuberculum sellae meningioma

Results were similar for the sensitivity analyses using study period instead of publication year, except for CSF leak: 7% (95%CI: 0–20) of patients from studies conducted between 2000 and 2005, compared with 13% (95%CI: 8–22%) between 2006 and 2010, and 3% (95%CI: 0–8%) between 2011 and 2015 (Fig. 3). Results did not differ for the sensitivity analyses only including case series describing patients with tuberculum sellae meningioma (Fig. 4), or olfactory groove meningioma, although the latter should be interpreted with caution as the number of studies and patients within some analyses is very small (Supplementary Figure 2).

**Fig. 5:**
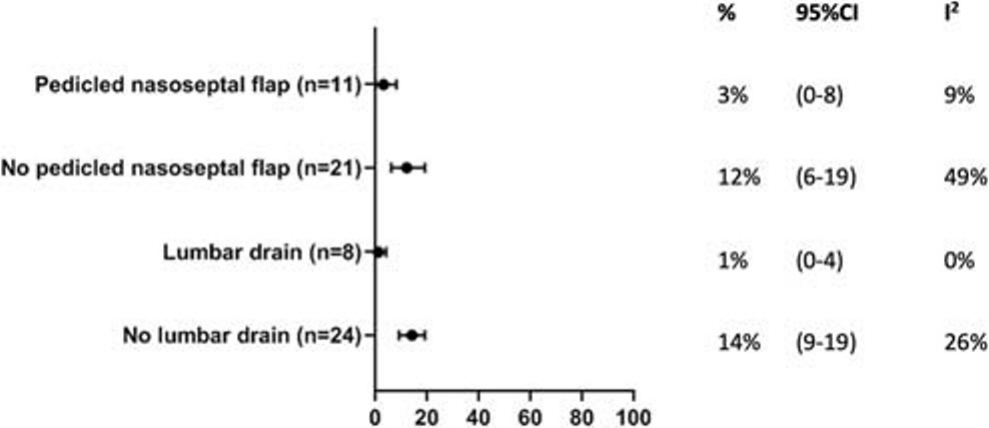
CSF leak in studies which clearly reported routine use of a pedicled nasoseptal flap, and which reported routine use of lumbar drains

In articles clearly describing the routine use of a pedicled nasoseptal flap, CSF leak was reported in 3% (95%CI: 0–8%) of patients, compared with 12% (95%: 6–19%) in those articles that did not describe routine use of a pedicled flap (Fig. 5). In articles describing the routine use of lumbar drains, CSF leak was reported in 1% (95%CI: 0–4%) of patients, compared with 14% (95%CI: 9–19%) in those articles that did not describe routine use of lumbar drains (Fig. 5). In the three articles describing routine use of the gasket seal closure technique, CSF leak was reported in 9% (95%CI: 0–46%). Note that all three studies were published by the same group [1, 2, 27].

## Discussion

Results of the main meta-analyses indicate that the percentage patients suffering from a CSF leak after extended endoscopic endonasal surgery for a tuberculum sellae or olfactory groove meningioma has decreased from 22% since publication of the first described case series to 4% in recent case series. Classifying studies on the actual described study period showed that the percentage CSF leak first increased and then decreased to percentages lower than the first published case series. We speculate this is because the first cases were highly selected and performed and described by very experienced endoscopic surgeons and pioneers of the extended endoscopic approach, while hereafter the approach found a broadened indication for use and was performed by an early majority of practitioners at various stages of their learning curve [12]. Gross total resection and improvement in visual function was achieved in approximately 85% of patients in all evaluated time periods. Similarly, outcomes of intraoperative arterial injury and mortality were stable over time, both outcomes occurring in almost no patients. These outcomes are fairly similar to meta-analyses of the transcranial approach for patients with tuberculum sellae and olfactory groove meningioma, with the exception that studies suggest that superior visual outcome might be achieved with the extended endoscopic approach in selected patients [5, 21, 22].

Compared with previously published meta-analyses, our results show indeed that the percentage CSF leak has decreased in the last decade with a 2011 analysis of anterior skull base meningioma reporting CSF leak in 32% of patients and a 2013 analysis of tuberculum sellae meningioma reporting CSF leak in 21% of patients [5, 32]. This improvement in the percentage CSF leak might be attributed to the development and improvement of new closure techniques, including the vascularized pedicled Hadad-Bassagasteguy flap, and the gasket seal closure technique [11, 18]. Due to its vascularization from the posterior sphenopalatine artery, the Hadad-Bassagasteguy flap is a fast healing flap with a large area coverage and large arc of rotation [11]. Its use as part of multilayer closure techniques, including synthetic materials, fat, and fascia lata, is adopted by many groups to decrease the chance of CSF leak [3, 4, 14, 26, 34]. Indeed, we describe that the percentage CSF leak in studies routinely using the Hadad-Bassagasteguy flap was 3%. In addition, multiple groups have published graded repair protocols, based on anticipated defect size and location, and intraoperative CSF leak grade to reduce unnecessary preparation of a pedicled nasoseptal flap, especially with the development of the rescue-flap [7, 19, 30]. Primarily described by the Cornell group, the gasket seal closure technique consisting of fascia lata and a bone buttress or other implant (e.g., MEDPOR) provides another technique for watertight closure of defects with excellent outcomes [18].

Standard use of lumbar drains to prevent CSF leaks is controversial as complications such as pneumocephalus and infections might not outweigh the potential benefit, especially as the percentage patients with a CSF leak has reduced with the development and improvement of closure techniques [23, 29]. However, a recent randomized controlled trial suggests that perioperative lumbar drain use combined with nasoseptal flap repair (in the context of dural defects > 1 cm^2^ and high flow intraop CSF leak), further decreases CSF rhinorrhea rates (21% vs 8%) without an increased risk of complications, such as infections [34]. Direct lumbar drain complications occurred in 4%, consisting of postoperative spinal headaches requiring a blood patch and retained catheter requiring no intervention [34]. These results suggest that the use of lumbar drains could play an important role in the prevention of CSF leaks in high risk cases, such as intradural meningioma resection [20, 33, 34]. The effectiveness of lumbar drains is underpinned in the current meta-analyses, as we report that CSF leak only occurred in 1% of patients in studies that routinely used a lumbar drain.

The decrease in CSF leak might also be attributed to a surgical learning curve. However, no clear improvement in the percentage patients with a gross total resection or improvement in visual outcomes was observed. This is in contrast with studies on the learning curve within a single large referral center, which showed improvement of both outcomes [17, 23]. Subcomponents of skull base surgery might demand particular surgical techniques, which run on different surgical curves [33]. In addition, different surgical groups, of whom the publications were analyzed in this meta-analyses, might be at different positions of their own respective learning curves. Regarding CSF leaks, it is actually described that a learning curve was only observed for complex skull base closure and closures of high-flow leaks, and not for small defects [23]. Similarly, for complex outcomes such as gross total resection and hormonal cure, a learning curve is described even after the first 200 cases, while not being described for surgical complications [33]. Unfortunately, the number of studies describing a center-specific learning curve is limited and could therefore not be analyzed separately in our study, limiting sound analyses of a potential surgical learning curve.

### Strengths and limitations

A limitation of this study is the small number of published studies with small, possibly highly selected, patient groups, and therefore selection bias and publication bias cannot be ruled out. However, to address selection bias to the best of our ability, we performed multiple sensitivity analyses which generally showed results in line with the main analyses, adding to the robustness of the results. Furthermore, we expect the possible impact of publication bias to be limited, as heterogeneity was seen in the reported outcomes, and asymmetry in the funnel plots was limited without any major changes in effect estimates using the trim and fill method. Nevertheless, the bar to submit and publish outcomes worse than the first reports, might have affected our outcomes. While we were able to perform analyses with studies routinely using a vascularized pedicled nasoseptal flap, we did not perform a separate analysis with studies routinely using and not using gasket seal closure techniques, as these studies were almost all from the same surgical group [1, 2, 27]. Furthermore, the analyses for patients with olfactory groove meningioma included a very limited number of studies, limiting the accuracy of the results and therefore readers are advised to interpret these results with caution. We acknowledge that our results might not be generalizable to patients with other pathologies than meningioma (e.g., chordoma, craniopharyngioma) as we chose to analyze a homogenous group of patients with tuberculum sellae and olfactory groove meningioma and did not include outcomes of other pathologies. Finally, analyses were performed on study-level, and therefore, we were not able to compare patients and tumor characteristics between publications. We encourage the international neurosurgical community to share individual patient-data for individual patient-data meta-analysis, which also provides results stratified for different tumor locations in more detail, enables analyses of outcomes currently rarely reported in literature, and may allow for comparison of the transcranial approach with the extended endoscopic approach.

## Conclusions

**Fig. 6:**
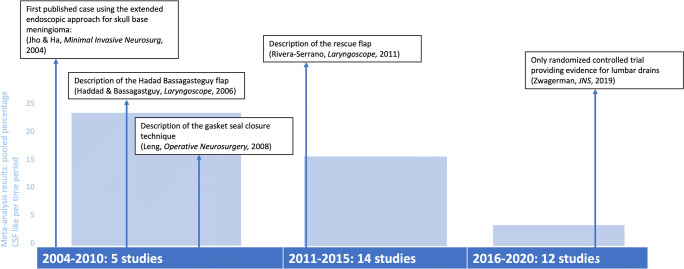
Timeline of selected key developments for the extended endoscopic skull base approach. Bars represent the pooled percentage CSF leak per time period”

We report a noticeable decrease in CSF leak over time, which might be attributed to the use of lumbar drains, development and improvement of new closure techniques (e.g., Hadad-Bassagasteguy flap, and gasket seal) and integration of these techniques within multilayer and graded repair protocols (Fig. 6). No improvement was observed for the percentage patients with a gross total resection, improvement in visual outcomes in those with preoperative deficits, intraoperative arterial injury, and 30-day mortality. An area for further research is understanding practice variations in skull base repair techniques and their corresponding CSF leak rates. Future multicenter studies aim to address this [16].

## Supplementary Information

ESM 1 (DOCX 607 kb)
