# Supplementary material for: Trends in cerebrospinal fluid leak rates following the extended endoscopic endonasal approach for anterior skull base meningioma: a meta-analysis over the last 20 years
Source: Acta Neurochir (Wien). 2020 Nov 14;163(3):711–9. doi: 10.1007/s00701-020-04641-x (PMC7886718; doi:10.1007/s00701-020-04641-x)
Supplement: Supplementary file 1 — (DOCX 607 kb) [file 701_2020_4641_MOESM1_ESM.docx]

**Supplementary Table 1: study characteristics**

| Author | Publication year | Hadad-flap | Gasket  seal | Drain | OGM | TSM | Age | % male | % WHO I | Mean size | Mean FU | Risk of bias  (mNOS) | % Gross toal resection | % visual improvement | % CSF leak | % arterial injury | % mortality |
| --- | --- | --- | --- | --- | --- | --- | --- | --- | --- | --- | --- | --- | --- | --- | --- | --- | --- |
| Cook | 2004 | No | no | no |  | 3 | 40 | 0% |  |  |  | 3 | 100% | 100% | 0% | 0% | 0% |
| De Devitiis | 2008 | No | no | no | 4 |  | 49 | 25% | 100% |  | 10 | 3 | 100% | 0% | 25% | 0% | 0% |
| De Devitiis | 2008 | No | no | no |  | 7 |  | 20% |  |  |  | 4 | 86% | 71% | 57% | 0% | 0% |
| Fatemi | 2009 | No | no | no |  | 14 | 51 | 29% |  |  | 27 | 6 | 50% | 82% | 29% | 0% | 0% |
| Wang | 2010 | No | no | no |  | 12 | 57 | 33% | 100% |  | 25 | 3 | 92% | 92% | 8% | 0% | 0% |
| Bowers | 2011 | No | no | no |  | 5 | 58 | 20% |  | 12 |  | 3 | 60% |  | 20% | 0% | 0% |
| Bohman | 2012 | yes | no | no |  | 5 | 53 | 40% |  |  | 12 | 4 | 80% | 80% | 20% | 0% | 0% |
| Chowdhury | 2012 | No | no | yes |  | 6 | 40 | 33% |  |  | 7 | 4 | 83% | 83% | 17% | 0% | 0% |
| Ogawa | 2012 | No | no | no |  | 19 | 59 | 26% | 89% |  | 36 | 3 | 79% | 74% | 5% | 0% | 0% |
| Padhye | 2012 | No | no | no | 8 |  | 52 | 25% | 100% | 41 |  | 3 | 88% | 25% | 38% | 0% | 0% |
| Padhye | 2012 | No | no | no |  | 3 | 66 | 0% | 100% | 9 | 3 | 3 | 100% | 100% | 0% | 0% | 0% |
| Gadgil | 2013 | yes | no | no |  | 5 | 51 | 40% | 1020% | 6 | 15 | 4 | 80% | 100% | 20% | 0% | 0% |
| Khan | 2014 | yes | no | no |  | 20 | 66 | 30% | 100% | 12 |  | 3 | 85% | 82% | 10% | 0% | 5% |
| Khan. | 2014 | yes | no | no | 15 |  |  |  | 67% |  |  | 4 | 82% | 80% | 7% | 0% | 0% |
| Koutourousiou | 2014 | no | no | no |  | 70 | 57 | 16% | 100% |  | 29 | 3 | 94% | 86% | 27% | 1% | 1% |
| Koutourousiou | 2014 | no | no | no | 50 |  | 57 | 36% | 20% |  | 33 | 3 | 67% | 93% | 30% | 2% | 0% |
| Al-meida | 2015 | no | no | no | 10 |  | 53 | 30% |  | 36 | 54 | 4 | 70% |  | 10% | 0% | 0% |
| Banu | 2015 | yes | yes | yes | 6 |  | 67 | 0% |  | 20 | 19 | 5 | 50% | 100% | 17% | 0% | 0% |
| Ceylan | 2015 | yes | no | no |  | 23 | 53 | 19% |  |  |  | 3 | 74% | 70% | 9% |  | 0% |
| Bander | 2016 | yes | yes | yes |  | 17 | 54 | 35% |  | 6 | 25 | 5 |  |  |  |  |  |
| Catapano | 2016 | yes | no | yes |  | 7 |  |  |  |  | 39 | 4 | 86% |  | 14% | 14% | 0% |
| Hayhurst | 2016 | no | no | no |  | 7 | 46 | 43% | 100% |  | 39 | 5 | 57% |  | 0% | 14% | 0% |
| Hayhurst | 2016 | no | no | no | 9 |  | 50 | 11% | 100% |  | 39 | 5 | 89% |  | 0% | 0% | 0% |
| Zoli | 2016 | no | no | no |  | 35 |  |  | 97% |  | 58 | 4 | 86% | 18% | 17% | 0% | 0% |
| Elshazly | 2017 | yes | no | no |  | 25 | 54 | 16% | 100% | 5 | 21 | 6 | 76% | 88% | 8% | 0% | 0% |
| Hayashi | 2017 | yes | no | yes |  | 22 | 58 | 32% |  |  |  | 4 | 68% | 83% | 0% | 0% | 0% |
| Linsler | 2017 | no | no | yes |  | 6 | 64 | 0% | 67% | 2 | 15 | 5 | 83% | 67% | 0% | 0% | 0% |
| Bernat | 2018 | yes | no | no |  | 20 | 59 |  |  |  | 38 | 5 | 0% |  | 0% | 0% | 0% |
| Bernat | 2018 | yes | no | no | 6 |  | 59 |  |  |  | 38 | 5 | 0% |  | 0% | 0% | 0% |
| Kong | 2018 | yes | no | yes |  | 84 | 54 | 24% |  |  | 28 | 5 | 83% | 85% | 5% | 0% | 1% |
| Kuga | 2018 | no | no | no |  | 7 | 54 | 0% |  | 3 | 19 | 6 | 100% | 100% | 14% | 0% | 0% |
| Liu 1 | 2018 | yes | no | no | 5 |  | 51 | 20% |  | 33 | 15 | 5 | 100% |  | 20% | 0% | 0% |
| Magill | 2018 | no | no | no |  | 44 |  |  |  |  | 46 | 5 |  |  | 11% |  | 0% |
| Ottenhausen | 2018 | no | yes | yes | 2 |  | 79 | 0% |  | 11 | 42 | 5 | 100% | 0% | 0% | 0% | 0% |
| Ottenhausen | 2018 | no | yes | yes |  | 30 | 57 | 37% |  | 7 | 42 | 5 |  |  |  |  |  |
| Song | 2018 | no | no | yes |  | 44 | 53 | 14% | 91% | 6 | 27 | 5 | 84% | 98% | 2% | 0% | 0% |

**Supplementary Figure 1: Raw and Trim & Fill funnel plots of the assessed outcomes**

**1) Gross total resection:**

| **Tuberculum Sellae Meningioma** | |
| --- | --- |
| Raw funnel plot | Trim & fill funnel plot |
| 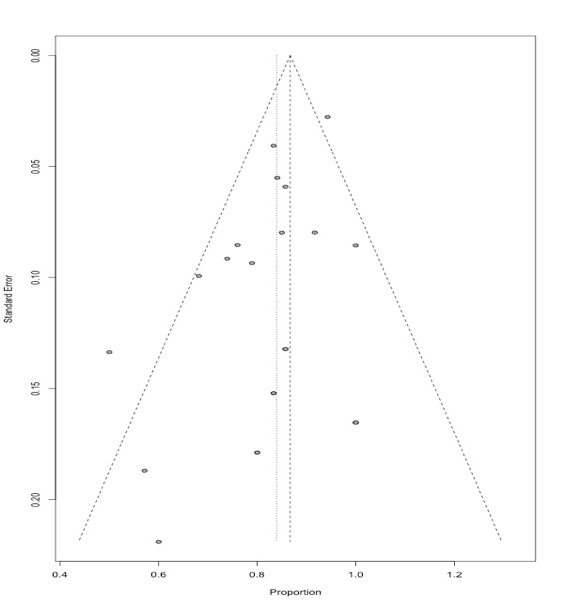 | 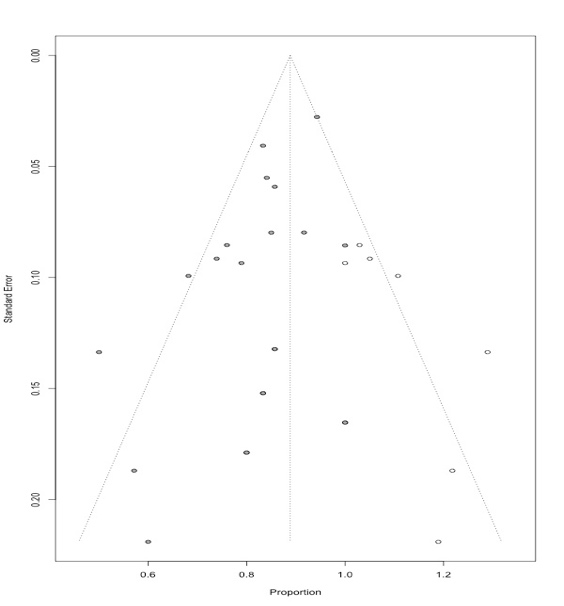 |
| **Olfactory Groove Meningioma** | |
| Raw funnel plot | Trim & fill funnel plot |
| 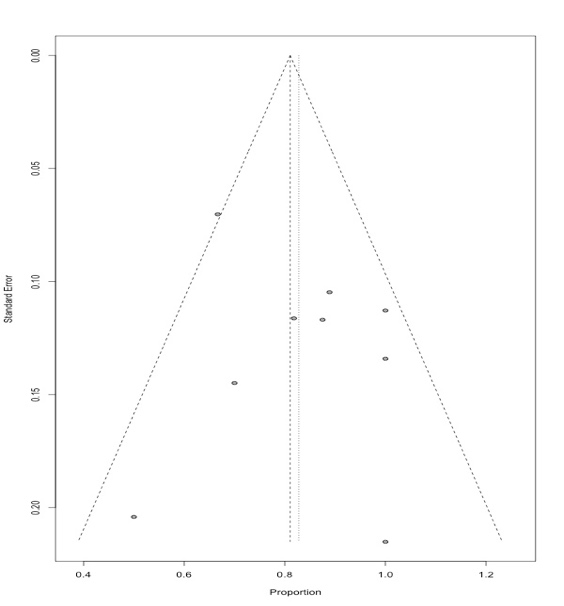 | 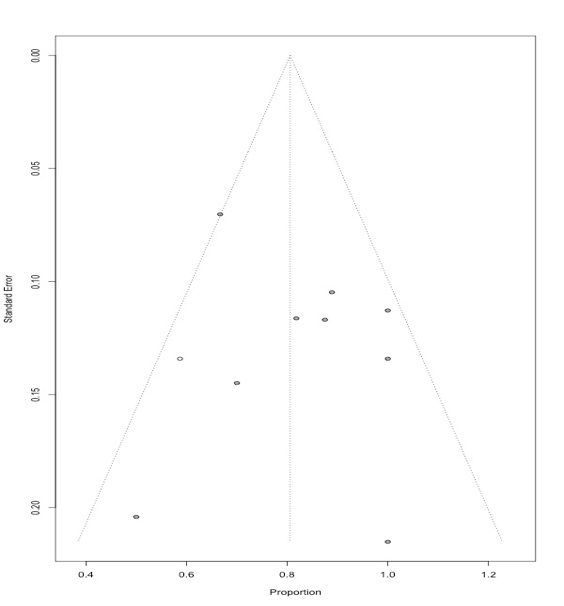 |

2) **Visual Improvement:**

| **Tuberculum Sellae Meningioma** | |
| --- | --- |
| Raw funnel plot | Trim & fill funnel plot |
| 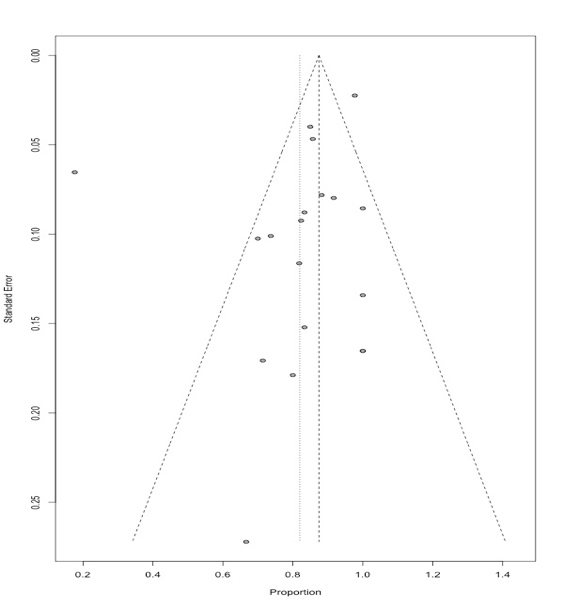 | 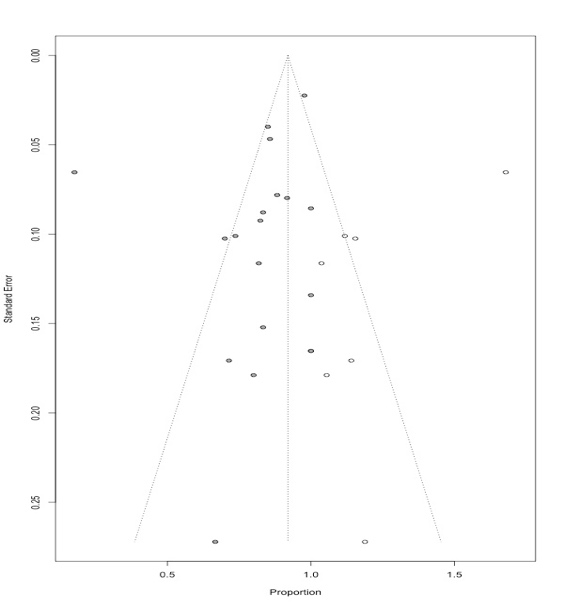 |
| **Olfactory Groove Meningioma** | |
| Raw funnel plot | Trim & fill funnel plot |
| 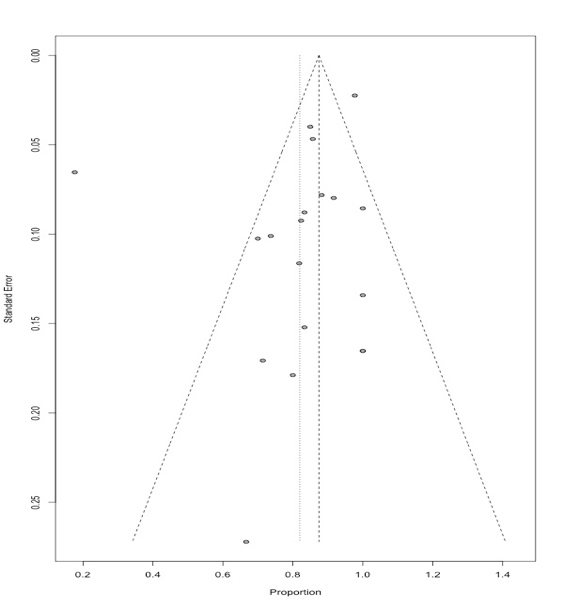 | 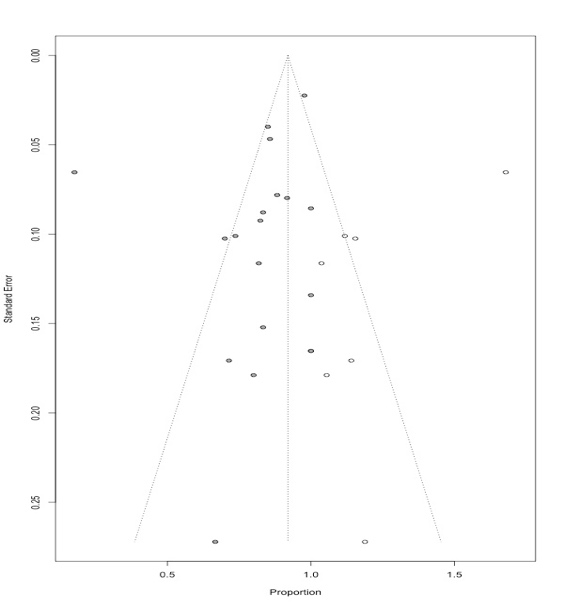 |

**3) Post-operative cerebrospinal fluid (CSF) leak**

| **Tuberculum Sellae Meningioma** | |
| --- | --- |
| Raw funnel plot | Trim & fill funnel plot |
| 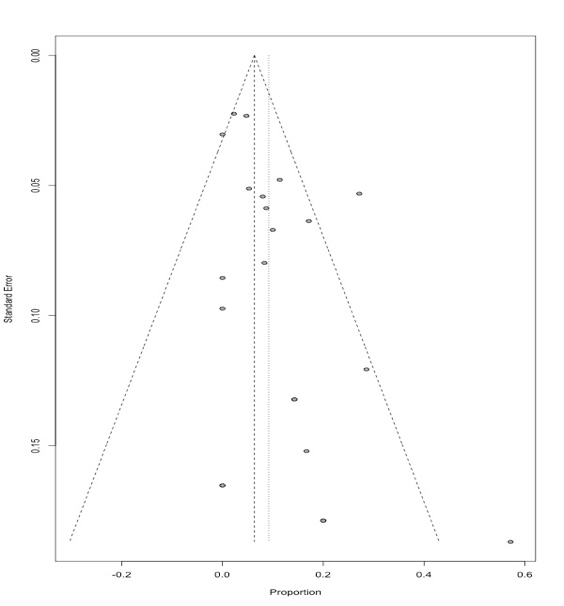 | 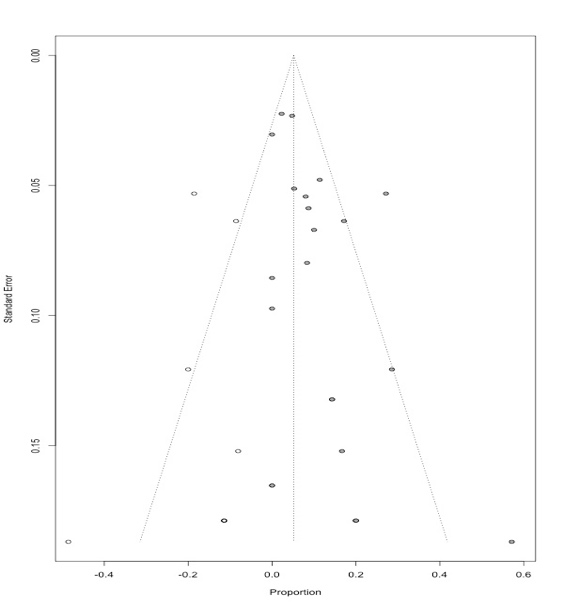 |
| **Olfactory Groove Meningioma** | |
| Raw funnel plot | Trim & fill funnel plot |
| 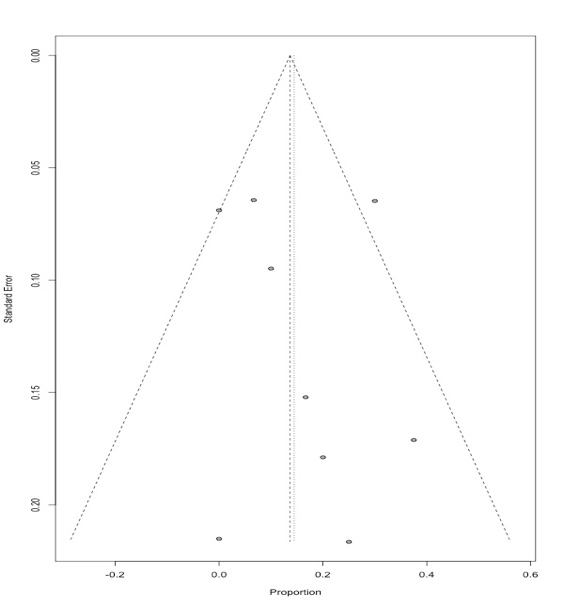 | 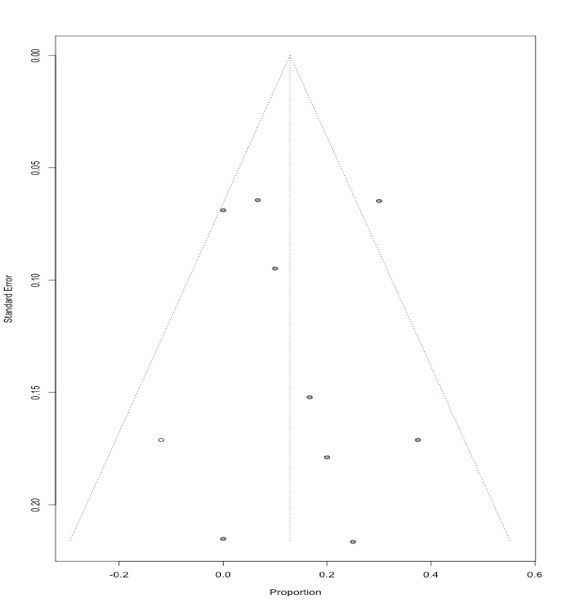 |

## 4) Intraoperative arterial injury:

| **Tuberculum Sellae Meningioma** | |
| --- | --- |
| Raw funnel plot | Trim & fill funnel plot |
| 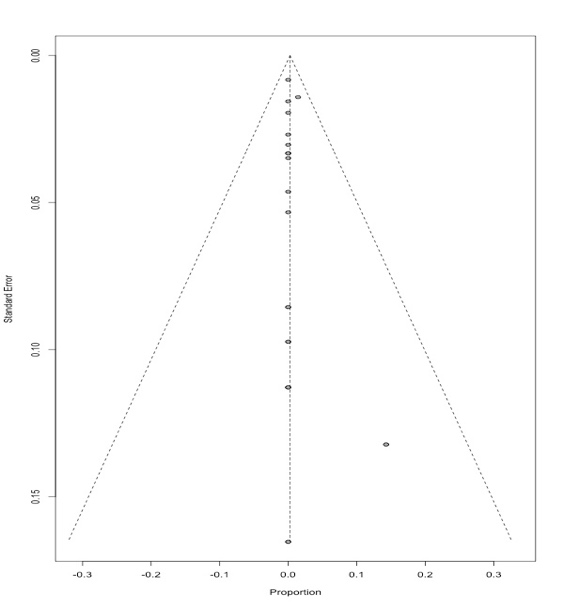 | 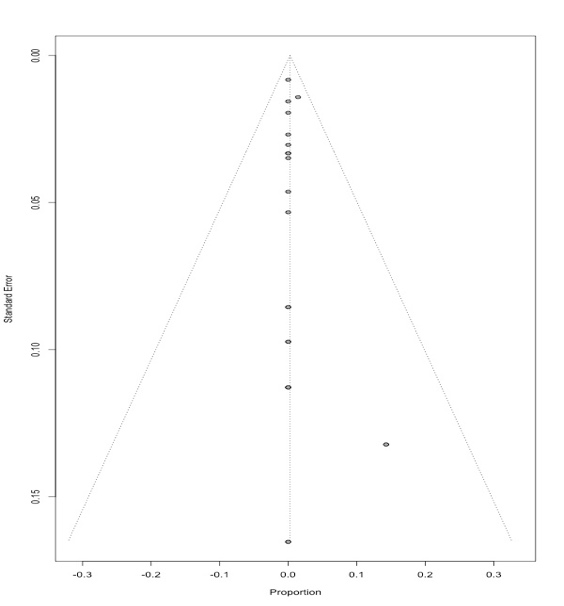 |
| **Olfactory Groove Meningioma** | |
| Raw funnel plot | Trim & fill funnel plot |
| 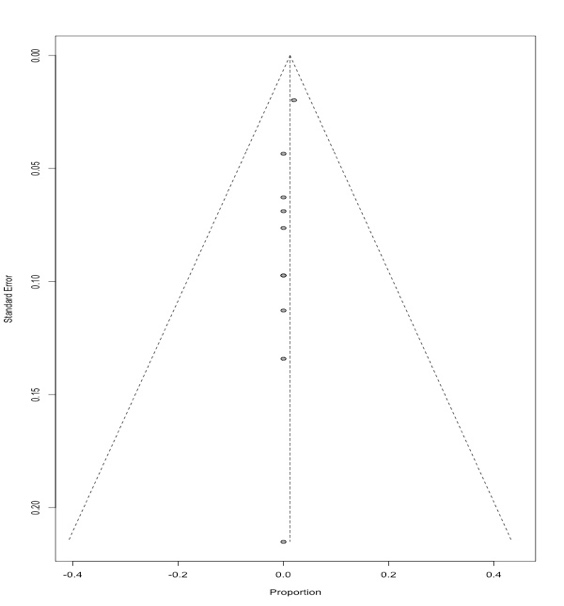 | 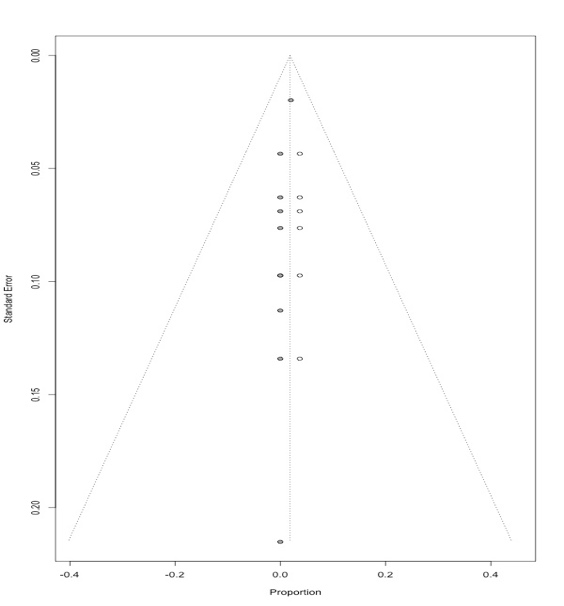 |

**5) 30-day mortality:**

| **Tuberculum Sellae Meningioma** | |
| --- | --- |
| Raw funnel plot | Trim & fill funnel plot |
| 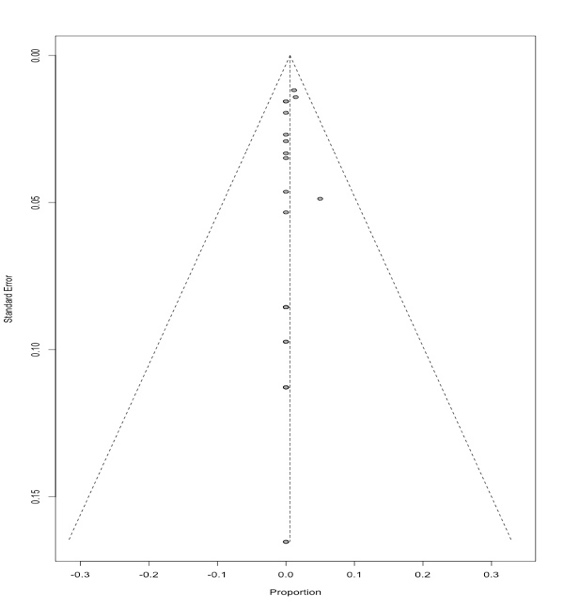 | 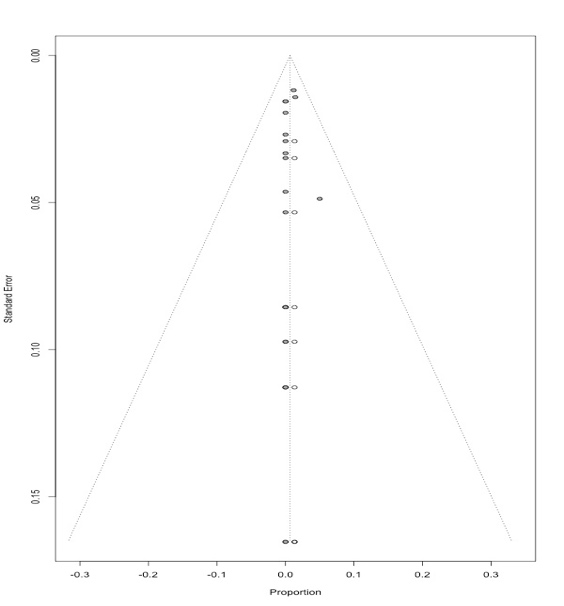 |
| **Olfactory Groove Meningioma** | |
| Raw funnel plot | Trim & fill funnel plot |
| 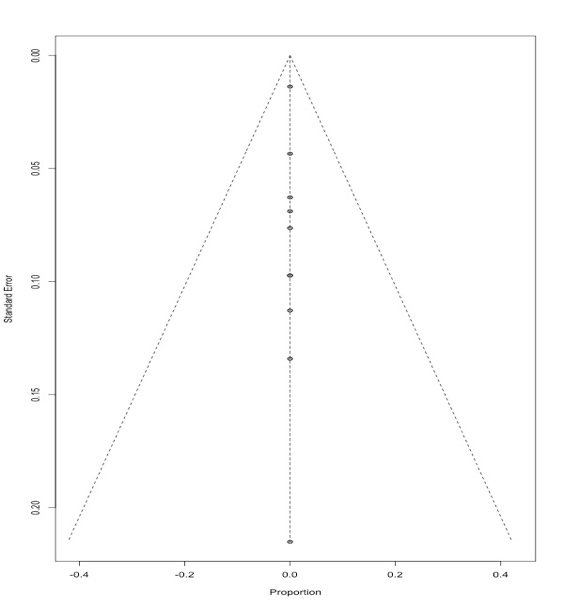 | 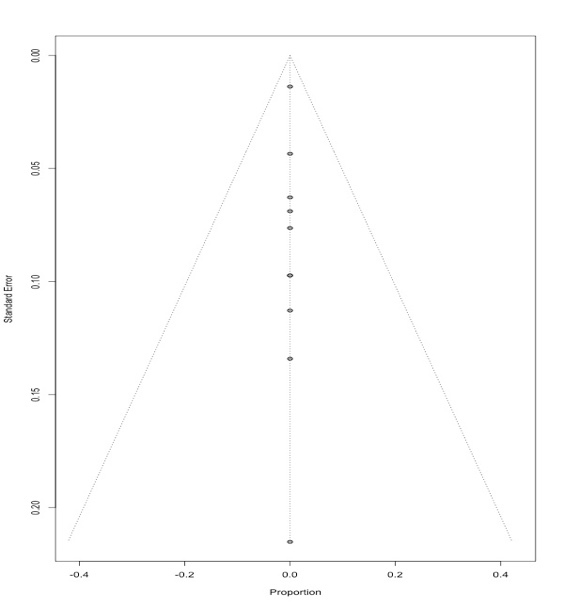 |

**Supplementary Figure 2: Results for patients with Olfactory groove meningioma**
